# High maternal self-efficacy is associated with meeting Institute of Medicine gestational weight gain recommendations

**DOI:** 10.1371/journal.pone.0226301

**Published:** 2019-12-11

**Authors:** Lyra Halili, Rebecca H. Liu, Ashley Weeks, Raywat Deonandan, Kristi B. Adamo

**Affiliations:** 1 School of Human Kinetics, Faculty of Health Sciences, University of Ottawa, Ottawa, Ontario, Canada; 2 Interdisciplinary School of Health Sciences, University of Ottawa, Ottawa, Ontario, Canada; Johns Hopkins University Bloomberg School of Public Health, UNITED STATES

## Abstract

**Objective:**

Fetal exposure to an intrauterine environment affected by maternal obesity and excessive gestational weight gain increases the likelihood of infants born large for gestational age and childhood obesity. This study examined behavioural factors and lifestyle practices associated with women’s perceived attainability of meeting the 2009 Institute of Medicine (IOM) weight gain guidelines.

**Methods:**

Cross-sectional data were collected from pregnant (n = 320) and postpartum (n = 1179) women who responded to the validated Canadian Electronic Maternal (EMat) health survey. Consenting women completed the survey through REDCap^™^ a secure, web-based data capture platform. Multiple logistic regression analyses were used to evaluate correlates associated with meeting or not meeting IOM recommendations. Odds ratios (ORs) were adjusted for relevant behavioural and sociodemographic covariates.

**Results:**

There were no significant differences between adjusted and unadjusted ORs for self-efficacy, barriers, and facilitators to weight gain during pregnancy. Women who reported *worry* regarding weight gain were significantly less likely to meet IOM guidelines (OR = 0.48, 95% CI = 0.33–0.69). Perceived controllability of behaviour was significantly associated with meeting IOM guidelines. An internal locus of control for weight gain was associated with an increased odds of meeting guidelines when women perceived to be in control of their weight gain (OR = 1.75, 95% CI = 1.29–2.37), healthy and exercised (OR = 1.91, 95% CI = 1.34–2.71), and when no barriers to healthy weight gain were perceived (OR = 1.43, 95% CI = 1.04–1.95); whereas, an external locus of control in which women viewed weight gain as beyond their control, was associated with a significantly reduced odds of achieving guidelines (OR = 0.58, 95% CI = 0.39–0.88).

**Conclusions:**

Self-efficacy and perceived controllability of behaviour are key factors to consider when developing pregnancy-specific interventions to help women achieve guideline-concordant weight gain and ensure the downstream health of both mother and infant.

## Introduction

In recent years, women of reproductive age are more likely to be overweight or obese [1,2]. In Canada, the prevalence of overweight and obesity has significantly increased among women of childbearing age [3] and approximately one third enter pregnancy at a body mass index (BMI) categorized as either overweight or obese [4]. In 2009, the American Institute of Medicine (IOM) established revised guidelines for weight gain during pregnancy to reflect the changing demographics and epidemiological profiles among obstetric populations. According to the IOM, women with a pre-pregnancy weight categorized as underweight (BMI < 18.5 kg/m^2^) are recommended to gain between 12.5 to 18.0 kg; women who are categorized as normal weight (BMI 18.5 to 24.9 kg/m^2^) are recommended to gain between 11.5 to 16.0 kg; those categorized as overweight (BMI 25.0 to 29.9 kg/m^2^) are recommended to gain between 7.0 to 11.5 kg; and lastly, women who are categorized as obese (all classes, BMI ≥ 30.0) are recommended to gain between 5.0 to 9.0 kg [5]. These guidelines, along with those from the Society of Obstetricians and Gynaecologists of Canada (SOGC) sought to mitigate adverse health consequences to both mother and child associated with excessive gestational weight gain (GWG) [5,6].

Entering pregnancy overweight or obese, or experiencing excessive GWG, contributes to an intergenerational cycle of obesity [7]. Women who give birth to large-for-gestational-age (LGA) neonates give rise to neonates that might follow a rapid growth trajectories, and if born female, would continue this cycle onto the next generation by also delivering large neonates who exhibit increased odds of downstream obesity [8,9]. Women who experience weight gain above guidelines and who are characterized as having a pre-pregnancy BMI of underweight or normal weight, can develop identical maternal-fetal health complications as women who are overweight or obese before pregnancy, indicating that excessive GWG is an independent and modifiable factor [10]. For mothers, GWG above IOM guidelines is associated with an increased risk of pre-eclampsia, cesarean delivery, gestational diabetes, postpartum weight retention, and obesity [8,11,12]. For infants, health risks include being born LGA, preterm delivery, downstream metabolic disorder, and overweight and obesity later in life [13].

Improving maternal and child health outcomes can be achieved by encouraging women to meet weight gain recommendations in combination with sound clinical guidance and support. Since its inception in 2009, approximately 50% of women continue to exceed IOM guidelines [14]. Although infrequently mentioned and often inaccurately used in clinical practice [15], awareness of IOM guidelines on weight gain, diet, and physical activity during pregnancy are essential contributors to healthy weight gain and overall prenatal health [16]. However, knowledge alone may not be sufficient to change prenatal behaviours [17]. Psychosocial factors associated with GWG such as self-perception have been found to play a role in excessive GWG [17] and should be considered. Furthermore, regular engagement in physical activity during pregnancy can be protective against excessive GWG [17–19], however, the psychosocial barriers and facilitators of physical activity during pregnancy have yet to be examined in detail.

To date, several studies have attempted to decipher the reasons why women gain weight outside of guidelines during pregnancy [17,20]. Recent systematic reviews of current interventions [21–23] have highlighted the importance of considering antecedent psychosocial risk factors of excessive GWG and encourage the integration of theory-based interventions in prenatal care to improve maternal health outcomes [24]. This is the first study to assess various psychosocial factors (self-efficacy, locus of control, self-confidence, knowledge, attitudes, perceptions, barriers, facilitators) associated with meeting IOM weight gain guidelines from the comprehensive and validated Canadian Electronic Maternal (EMat) health survey. The primary purpose of this study was to examine the association between psychosocial factors and achieving guideline-concordant weight gain during pregnancy. A secondary purpose was to examine the association between physical activity practices, barriers, and facilitators to exercise, and achieving IOM-recommended weight gain during pregnancy.

## Methods

### Ethical approval

The current study was performed in accordance with the regulations set forth by the Declaration of Helsinki. The University of Ottawa, Research Ethics Board (file number: A06-15-02) and the Children’s Hospital of Eastern Ontario Research Institute, Research Ethics Board (file number: CHEOREB# 14/183X) approved the ethical components of the present study. Participants provided consent to publish. Electronic and informed consent was obtained from all survey respondents before commencing the survey.

### Study design

Cross-sectional data were gathered from both currently pregnant women and women who gave birth in the years following the adoption of the 2009 IOM guidelines (i.e. less than or equal to 5 years postpartum). The EMat health survey, a validated questionnaire designed to assess women’s knowledge and perceptions of current IOM gestational weight gain guidelines, physical activity, and nutritional practices, as well as pregnancy-related health behaviours, is hosted online through the Research Electronic Data Capture, REDCap^™^ (Vanderbilt University, Nashville, TN, USA), a secure, web-based software platform designed to support data capture for research studies [25,26]. Consenting participants securely and confidentially took part in the online survey.

### Survey development

The survey, grounded in Social Cognitive Theory, was constructed using branching logic to prevent redundancies and was developed in eight comprehensive steps by Ockenden and colleagues [27]. The authors from the cited validation manuscript initially examined 110 items for the questionnaire (50 for current pregnancy and 60 for past pregnancy). After removing duplicate and ambiguous items, the final version of the questionnaire consisted of two sections with six constructs that included a total of 60 items. Section 1 of the questionnaire was ‘Inclusion/Exclusion’ and section 2 was ‘Demographics’. The six constructs included were (1) Health Practices, (2) Pregnancy Weight, (3) Physical Activity, (4) Diet, (5), Pregnancy Intentions and/or Pregnancy Practices, and (6) Diet and Weight Gain Perceptions. The reliability scores for most constructs were considered moderate to high with the highest score observed for the Health Practices construct. Content validity was assessed by an expert panel of seven individuals including a clinical psychologist specializing in health behaviour, a qualitative researcher in women’s health with expertise in socio-cultural factors of behaviour, a physiologist with expertise in nutrition and physical activity during pregnancy, an internal medicine and obstetrics specialist, two maternal health experts, and a pregnant woman who was also a recent mother. Test-retest reliability was assessed using a sample of 39 individuals from the target population (pregnant and recently pregnant women) over a 14-day interval. Preliminary validation of the survey concluded strong test-retest reliability indicating confidence in this questionnaire for the target population. All items that showed statistically significant (*p* < 0.05) test-retest reliability remained in the final questionnaire.

### Survey respondents and administration

Pregnant and postpartum women who gave birth after May 2009 were invited to participate in the study. Survey responses were collected over four years from 2014 to 2018. Women were recruited through social media websites (Facebook and Twitter postings) and the corresponding author’s laboratory website. A snowball recruitment method was used in which participants who completed the study promoted the survey to their friends, families, and colleagues and provided them with the information to access the survey. Women who agreed to participate were directed to a secure link where they could access the survey. Once the link was selected, women were taken to the electronic consent form and could only proceed to the questions after providing informed consent. The survey was limited to English-speaking pregnant and postpartum women only, who were at least 18 years of age.

### Variables of interest

The dependent variable of the study was whether pregnant and postpartum women met the 2009 IOM gestational weight gain recommendations. Independent variables included in the analysis were composed of questions associated with self-efficacy (addressing both internal and external locus of control), self-confidence, barriers/facilitators to meeting a target weight during pregnancy and adopting healthful lifestyle behaviours. Participants were asked about physical activity practices and healthy eating during pregnancy. Exploratory variables included in the analysis were comprised of questions associated with barriers and facilitators to adopting these healthful lifestyle behaviours.

### Behavioural and sociodemographic covariates

Behavioural covariates included smoking frequency and alcohol consumption throughout pregnancy using the following selection options: Every day, 2–3 times a week, once a week, once every 2–3 weeks, once a month, on special occasions only, never, and prefer not to answer. Sociodemographic covariates such as age, ethnicity, education, employment status, income, marital status, and geographical living environment were also included in the analysis as potential confounders.

### Statistical analysis

Among completed responses from pregnant women, self-reported pre-pregnancy weight (6 months prior) and height were used to calculate pre-pregnancy BMI. Pregnant women’s (*n* = 320) current gestational age (weeks) and weight (kg) were used to compute whether respondents were on track to meeting guidelines. Perceived pre-pregnancy BMI category (1-year prior) from postpartum women (*n* = 1179) along with self-reported weight gained during the most recent past pregnancy was used to determine whether women met GWG recommendations. Data from both pregnant and postpartum women were combined in the analyses for questions related to self-efficacy, barriers and facilitators to weight gain as these questions were programmed to be asked to all women who took part in the survey. Multiple logistic regression analyses were performed using IBM SPPS ver. 24 (IBM Inc., Armonk, NY, USA). To determine whether women met IOM guidelines, pre-pregnancy BMI and weight gained during pregnancy were coded and computed using SAS ver. 9.4 (SAS Institute, Cary, NC, USA). Women were categorized as having met guidelines or not met guidelines. Guideline accordance served as the dichotomous outcome (dependent) variable described above. For all data analyses, statistical significance was defined at *p* ≤ 0.05.

## Results

Overall, 1488 women (both pregnant and postpartum women) completed the survey. Missing and incomplete data constituted a total of 357 participants, thus responses from 1130 participants were included in the analysis. The characteristics of survey respondents are found in Table 1. Most women were between the ages of 30 to 39 years (pregnant *n* = 222, 69.4%; postpartum *n* = 874, 74.1%), married (pregnant *n* = 256, 80.0%; postpartum *n* = 992, 84.3%), and had graduate degrees (pregnant *n* = 122, 38.1%; postpartum *n* = 512, 43.5%). Pregnancy-related characteristics of respondents are presented in Table 2. The proportion of pregnant and postpartum women who met IOM guidelines (pregnant *n* = 158, 49.4%; postpartum *n* = 534, 45.3%) was lower than those who did not meet guidelines (pregnant *n* = 162, 50.6%; postpartum *n* = 645, 54.7%).

**Table 1:** Characteristics of survey respondents.

Characteristic	Currently Pregnant (n, %)	Postpartum (n, %)
**Total**	**320 (21)**	**1179 (79)**
*Age Group*		
18 to 29	88 (27.5)	185 (15.7)
30 to 39	222 (69.4)	874 (74.1)
40 to 50+	10 (3.1)	120 (10.2)
*Marital Status*		
Married/In a relationship	310 (96.9)	1132 (96.0)
Not married	9 (2.8)	45 (3.8)
*Highest Level of Education*		
Less than High School	0 (0.0)	2 (0.2)
High School/GED	13 (4.1)	31 (2.6)
Some Post-Secondary Education	12 (3.8)	59 (5.0)
Trade Certification or Diploma	7 (2.2)	27 (2.3)
Non-University Certificate or Diploma	53 (16.6)	152 (12.9)
Bachelor’s Degree	113 (35.3)	394 (33.5)
Graduate Degree	122 (38.1)	512 (43.5)
*Employment Status*		
Employed	285 (89.1)	1010 (85.7)
Currently unemployed	35 (10.9)	168 (14.2)
*Approximate Household Income*		
Less than $30,000 –$60,000	33 (10.3)	134 (11.4)
$60,000 –$120,000	133 (41.6)	400 (34.0)
$120,000 –$150,000	137 (42.8)	587 (49.9)
Prefer not to answer	17 (5.3)	55 (4.7)
*Living Environment*		
Urban area	107 (33.4)	386 (32.7)
Suburban	152 (47.5)	587 (49.8)
Rural area	61 (19.1)	206 (17.5)

GED, General Equivalency Diploma.

Note. An urban area is defined as a large city (population greater than 1 million); a suburban area is defined as a smaller city or region outside an urban area (population greater than 10,000 to less than 1 million); and a rural area is defined as a small town (population less than 10,000).

**Table 2:** Pregnancy-related characteristics of survey respondents.

Characteristic	Currently Pregnant (*n*, %)	Postpartum (*n*, %)
**Total**	**n = 320 (21)**	**n = 1179 (79)**
*IOM GWG Guidelines*		
Met guidelines	158 (49.4)	534 (45.3)
Did not meet guidelines	162 (50.6)	645 (54.7)
*Country of Prenatal Care*		
Canada	310 (96.9)	1112 (95.0)
USA	4 (1.3)	24 (2.1)
Other	6 (1.9)	35 (3.0)
*Number of Biological Children under 6 years old at home*		
None	4 (1.8)	34 (2.9)
1	160 (71.4)	680 (57.7)
2 or more	60 (26.8)	464 (39.4)
*Pregnancy planned*		
Yes, pregnancy occurred naturally	267 (83.4)	954 (81.3)
Yes, pregnancy occurred due to fertility treatment	22 (6.9)	80 (6.8)
No, pregnancy was not planned	29 (9.1)	137 (11.7)
*Perceived Weight Category*		
Underweight	5 (1.6)	33 (2.8)
Normal weight	213 (67.0)	807 (68.9)
Overweight	79 (24.8)	275 (23.5)
Obese	20 (6.3)	57 (4.9)
I don’t know	1 (0.3)	0 (0.0)
Prefer not to answer	0 (0.0)	0 (0.0)
*Do you know your personal calorie requirements*?		
Yes	163 (53.1)	673 (62.1)
No	144 (46.9)	411 (37.9)
*Have/did your eating habits change during pregnancy*?		
My eating habits are healthier	46 (15.0)	286 (25.9)
My eating habits have stayed the same	185 (60.3)	531 (48.1)
My eating habits are less healthy	68 (22.2)	269 (24.4)
I don’t know	8 (2.6)	17 (1.5)
*Do you consider feelings of weight dissatisfaction after pregnancy normal within your social network*?		
Yes	52 (16.3)	846 (72.3)
No	65 (20.3)	192 (16.4)
Don't know	23 (7.2)	130 (11.1)
Prefer not to answer	2 (0.6)	3 (0.3)

GWG, Gestational Weight Gain; IOM, Institute of Medicine.

Several correlates were significantly associated with meeting or not meeting recommendations. Combined analyses of pregnant and postpartum women (Table 3; *n* = 1131) found that women who reported they *worried* about gaining too much weight while pregnant were 52% less likely to meet IOM weight gain guidelines relative to those who reported they did not worry (OR = 0.48, 95% CI = 0.33–0.69). Women who agreed with the statement, “*Do you think women should be careful about gaining too much weight”*, were approximately twice as likely to meet weight gain guidelines than those who disagreed with the statement (OR = 1.75, 95% CI = 1.07–2.85). Those who presented with an internal locus of control in which they agreed to the following three statements, “*I can control the amount of weight I gain”* (OR = 1.75, 95% CI = 1.29–2.37), *“If I am healthy and exercise*, *I can control my weight”* (OR = 1.91, 95% CI = 1.34–2.71) and, “*I don’t believe there are any barriers to gaining a healthy amount of weight*,” (OR = 1.43, 95% CI = 1.04–1.95) were all also approximately twice as likely to meet IOM guidelines whereas those who presented with an external locus of control and perceived that “*Weight gain is beyond control of the mother”* were 42% less likely to meet weight gain recommendations (OR = 0.58, 95% CI = 0.39–0.88). There were no statistically significant associations among perceived facilitators towards gaining a targeted weight while pregnant and meeting IOM guidelines.

**Table 3:** Self-efficacy, barriers, and facilitators to weight gain among pregnant and postpartum women.

Correlates	Adjusted OR (95% CI)
*Do/Did you worry that you may gain too much weight*?	
No	1.00
Sometimes	0.77 (0.55–1.07)
Yes	**0.48 (0.33–0.69)**
*Do/Did you feel it acceptable to gain as much weight as you want*?	
Disagree	1.00
Not sure	*0*.*64 (0*.*40–1*.*03)*
Agree	0.94 (0.63–1.40)
*If you are eating a well-balanced diet*, *do you feel it shouldn't matter how much weight you gain*?	
Disagree	1.00
Not sure	0.81 (0.55–1.19)
Agree	*0*.*83 (0*.*63–1*.*09)*
*Do you think women should be careful about gaining too much weight*?	
Disagree	1.00
Not sure	**1.87 (1.04–3.35)**
Agree	**1.75 (1.07–2.85)**
*I can control the amount of weight I gain*.	
Disagree	1.00
Not sure	1.27 (0.89–1.81)
Agree	**1.75 (1.29–2.37)**
*If I am healthy and exercise*, *I can control my weight*.	
Disagree	1.00
Not sure	**1.69 (1.12–2.53)**
Agree	**1.91 (1.34–2.71)**
*Although you may have many obligations*, *do you feel that you can still be physically active*?	
No (n = 198)	0.93 (0.57–1.53)
Yes (n = 930)	1.00 (0.67–1.50)
*Do you feel that you can eat healthy foods and avoid foods that aren't good for you*?	
No (n = 169)	*0*.*63 (0*.*38–1*.*05)*
Yes (n = 953)	0.96 (0.65–1.44)
*What do you believe are the barriers to gaining within a targeted weight*?	
Lack of support from family or friends	*1*.*32 (0*.*96–1*.*82)*
Lack of guidance from HCP	1.33 (1.00–1.78)
Weight gain is beyond control of the mother	**0.58 (0.39–0.88)**
It is difficult to exercise	0.88 (0.68–1.14)
It is difficult to eat healthy	0.87 (0.63–1.22)
I don't believe there are any barriers to gaining a healthy amount of weight	**1.43 (1.04–1.95)**
*What do you believe contributes to women being able to gain within a targeted weight*?	
Support from family or friends	1.00 (0.78–1.28)
Guidance from HCP	1.08 (0.85–1.37)
Weight gain management is a priority for me	1.04 (0.79–1.37)
I get regular exercise	0.94 (0.74–1.20)
I eat healthy	1.02 (0.80–1.31)

CI, Confidence Interval; HCP, Health Care Practitioner; OR, Odds Ratio.

Note. Total Pregnant and Postpartum (n = 1488); Missing and Incomplete (n = 357); Included in analysis (n = 1131); did not meet guidelines (n = 611); met guidelines (n = 520). ORs were adjusted for relevant behavioural and sociodemographic covariates. There were no significant differences between adjusted and unadjusted ORs. Statistically significant associations are **bolded** (*p* < 0.05). Associations approaching significance are *italicized*.

Presented in Table 4 are physical activity practices, barriers, and facilitators to exercise. Overall, most pregnant (n = 263, 84.0%) and postpartum (n = 918, 80.2%) women reported planning to exercise during pregnancy, however, when asked whether they engaged in exercise as much as they would have liked during pregnancy, the majority of pregnant (n = 248, 77.5%) and postpartum (n = 788, 66.8%) women stated “No”. Pregnant women who selected “too tired” as a barrier to not exercising as much as they would like were approximately 50% less likely to meet IOM guidelines (OR = 0.53, 95% CI = 0.27–0.95). Additionally, pregnant women who selected “too much pain/discomfort” (OR = 0.17, 95% CI = 0.08–0.35) and “too self-conscious” (OR = 0.07, 95% CI = 0.01–0.63) were approximately 90% less likely to meet weight gain recommendations. Among postpartum women, social support was a perceived barrier to meeting IOM guidelines whereby those who indicated that they had “no one to go with” (OR = 0.39, 95% CI = 0.19–0.80) were significantly less likely to meet weight gain recommendations.

**Table 4:** Physical activity practices, barriers, and facilitators to exercise.

Variables	Pregnant Adjusted OR (95% CI)	Postpartum Adjusted OR (95% CI)
**Physical activity practices, barriers and facilitators to exercise**		
*Do/did you plan to exercise*?	n = 320	n = 1179
No	50 (16.0%)	226 (19.8%)
Yes	263 (84.0%)	918 (80.2%)
*Did you exercise as much as you would like*?		
No	248 (77.5%)	788 (66.8%)
Yes	65 (20.3%)	373 (31.6%)
*Why have you not exercised as much as you would like*?		
Unsure of what to do	0.66 (0.25–1.77)	0.84 (0.51–1.38)
Told not to exercise by HCP (health concerns)	0.62 (0.17–2.17)	0.76 (0.47–1.23)
Too tired	**0.53 (0.27–0.95)**	0.83 (0.65–1.06)
Felt sick	0.73 (0.41–1.30)	0.90 (0.68–1.20)
Too much pain/discomfort	**0.17 (0.08–0.35)**	*0*.*77 (0*.*57–1*.*06)*
Lack of time	0.81 (0.45–1.47)	1.03 (0.79–1.33)
Lack of money	1.37 (0.16–11.8)	0.74 (0.34–1.61)
Difficulty obtaining childcare	1.25 (0.55–2.81)	*0*.*73 (0*.*49–1*.*10)*
No one to go with	1.79 (0.39–8.22)	**0.39 (0.19–0.80)**
No interest/motivation	2.08 (0.94–4.60)	0.96 (0.66–1.39)
Too self-conscious	**0.07 (0.01–0.63)**	*0*.*47 (0*.*20–1*.*10)*
Illness	0.67 (0.14–3.31)	0.76 (0.40–1.42)
*What do you believe has helped you exercise*?		
I am active person	1.14 (0.50–2.59)	*1*.*26 (0*.*94–1*.*69)*
I felt fine (no sickness or fatigue)	1.00 (0.33–2.95)	*1*.*38 (0*.*99–1*.*93)*
I had/ have support from family	*3*.*48 (0*.*94–12*.*9)*	1.09 (0.74–1.61)
I had/ have support from friends	1.37 (0.19–9.80)	1.48 (0.86–2.55)
I had the support of my employer	0.89 (0.15–5.13)	1.03 (0.48–2.25)
*What are your motivations for exercise*?		
Maintain/limit weight gain	0.93 (0.53–1.62)	1.04 (0.82–1.34)
Socialize	1.32 (0.65–2.67)	1.14 (0.83–1.57)
Competition	4.21 (0.41–43.4)	2.48 (1.00–6.14)
Release tension	0.73 (0.42–1.27)	0.90 (0.69–1.17)
Enhance mental health	0.75 (0.43–1.31)	0.97 (0.75–1.25)
Keep fit	0.88 (0.50–1.54)	1.11 (0.86–1.44)
For the health of my baby	1.07 (0.58–1.98)	0.97 (0.75–1.26)
For my health	1.14 (0.56–2.31)	0.99 (0.75–1.31)

CI, Confidence Interval; HCP, Health Care Practitioner; OR, Odds Ratio.

Note. Statistically significant associations are **bolded** (*p* < 0.05). Associations approaching significance are *italicized*.

## Discussion

The purpose of this study was to examine the psychosocial factors (self-efficacy and perceived controllability of behaviours), along with the barriers and facilitators to physical activity practices during pregnancy and their associations with meeting IOM weight gain recommendations. We found that high self-efficacy and internal locus of control were associated with a greater likelihood of achieving guideline-concordant weight gain, whereas low self-efficacy and external locus of control were associated with a lower likelihood of adhering to weight gain recommendations. Self-efficacy refers to the belief in one’s capacity to exercise control over challenging situations wherein low self-efficacy is associated with helplessness and high self-efficacy is associated with competence and successful outcomes [28,29]. Perceived controllability or locus of control towards one’s actions is related to self-efficacy, yet there are key distinctions. Individuals with an internal locus of control believe that the consequences of their actions are due to their abilities, whereas individuals with an external locus of control believe that consequences are a result of factors outside of their control [30]. These associations continued to be significant even after adjusting for relevant sociodemographic covariates. Women who agreed that *care* for weight gain should be considered during pregnancy were nearly twice as likely to meet guidelines, demonstrating greater self-awareness and knowledge of recommendations. In contrast, weight-related concern or *worry* resulted in reduced odds of meeting weight gain guidelines.

A woman’s perception of self and sense of empowerment may impact maternal weight gain during pregnancy [17]. Our findings support the notion that women who were concerned about gaining too much weight while pregnant were significantly less likely to meet IOM weight gain recommendations. Furthermore, our finding regarding weight-related concern or worry is in line with a recent systematic review which demonstrated that some risk factors for excessive GWG include concern about weight gain, negative attitudes towards weight gain and inaccurate perceptions about weight [21]. Previous studies have found that concern about weight gain is a risk factor for excessive GWG [31,32]. However, studies in this area of research are limited. There is evidence to suggest that maternal pregnancy stress influences GWG through an elevation of cortisol levels [33]. In fact, a study examining a stress reduction intervention to help promote healthy GWG found that women were highly interested in these interventions involving a variety of mindfulness techniques [34]. Stress or worry have been considered modifiable psychosocial risk factors; given that our findings demonstrate that worry is associated with weight gain, this risk factor should be taken into account when designing future interventions to help reduce the likelihood of excessive GWG, ultimately improving weight and health trajectories for both mother and infant.

In addition to perceived worry, pregnant and postpartum women’s attitude towards weight gain was equally significant in predicting IOM guideline-concordant weight gain. Our data illustrated that women who viewed that care should be taken in gaining too much weight while pregnant were twice as likely to meet IOM recommendations than those who failed to recognize this as a valuable concern throughout pregnancy. Although we cannot conclude with certainty, this attitude towards weight gain may be indicative of pregnant and postpartum women’s knowledge of the health impact associated with excessive GWG. This finding is in contrast to a systematic review and meta-synthesis of 42 qualitative empirical research studies conducted in high-income countries and published between 2005 and 2015 [35], which highlighted that women are motivated to improve fetal health but they may not necessarily be aware of the link between gaining excess weight during pregnancy and negative fetal health implications. The discrepancies between our findings and these results may be due to the methodological differences (quantitative versus qualitative) in research design along with differences in the level of education and socioeconomic status in our sample population. Importantly, our finding regarding care towards weight gain during pregnancy can be explained by well-versed psychological concepts from the Theory of Planned Behaviour (TPB) [36], whereby an attitude (i.e. care should be taken) towards a certain behaviour (i.e. too much weight gain during pregnancy) results in behavioural changes (i.e. monitoring weight gain, therefore greater odds of meeting guidelines). Previous work has found the TPB to be a useful framework in assessing women’s weight-related intentions throughout pregnancy [37,38]. Therefore, this attitude towards weight gain leading to an increased likelihood of meeting IOM recommendations supports the contention that the understanding of women’s perceptions during pregnancy can be used to inform future behavioural interventions.

The Electronic Maternal Health Survey’s incorporation of Social Cognitive Theory (SCT) during development allows for identification of pregnant and postpartum women most at risk of experiencing excessive GWG or guideline-discordant weight gain. The SCT takes into consideration personal, behavioural, and environmental interactions that ultimately influence outcome behaviours [39]. Pregnant and postpartum women with a high self-efficacy, that is, their success in meeting IOM guidelines was attributed to an internal locus of control and associated with a significantly higher odds of meeting weight gain recommendations. In parallel to our current study, previous work has shown that high self-efficacy towards healthy weight gain during pregnancy served a protective role against excessive GWG [40]. Moreover, a study examining weight-related self-efficacy concerning maternal body weight between early pregnancy and two years postpartum found that high self-efficacy towards weight control was associated with lower maternal body weight [41].

Along with our findings regarding the internal locus of control, low self-efficacy, and the external locus of control, whereby the success of meeting IOM recommendations was attributed to factors beyond the individuals’ control, was found to be significantly associated with a reduced odds of meeting guidelines. This finding is partially supported by a previous study examining the psychosocial determinants of GWG adequacy using a unique tool that assessed women’s perceived locus of control over fetal health. The researchers found that women who believed that external factors and chance were associated with fetal health were more likely to deviate from GWG guidelines [42]. To date, however, few studies have examined weight-related self-efficacy and locus of control as they relate to meeting IOM gestational weight gain guidelines, particularly in a predominantly Canadian sample. Our findings regarding self-efficacy and locus of control with reference to meeting IOM recommendations further contribute to current literature and encourage future research on how an internal locus of control for weight gain during pregnancy can be used to achieve healthful pregnancy outcomes.

Although not statistically significant, it is important to acknowledge that pregnant and postpartum women who reported that they did not feel they could eat healthy foods and avoid foods that are not good for them, demonstrated a tendency towards a reduced odds of meeting IOM recommendations. In a study assessing pregnant Australian women’s knowledge of appropriate GWG and dietary guidelines, women demonstrated an adequate level of broad knowledge of dietary recommendations, but their detailed knowledge in this area was poor [43]. Notably, poor maternal nutritional practices have been associated with postpartum weight retention and excessive GWG [44], thereby contributing to an intergenerational cycle of obesity [45]. Given our current understanding of the health ramifications associated with a cross-generational cycle of obesity, women continue to foster inaccurate beliefs of nutritional requirements during pregnancy and report minimal counseling from their care providers [15]. Thus, this may partially explain why women who reported a lack of an ability to control their eating habits and nutritional practices throughout pregnancy, presented with a tendency (OR = 0.63, *p* = 0.08) towards not achieving IOM weight gain guidelines.

Physical activity, similar to adopting healthy dietary practices during pregnancy, is paramount to achieving appropriate GWG and numerous short- and long-term health benefits to both the mother and developing fetus. The SOGC and the American College of Obstetricians and Gynecologists (ACOG) recommend that women with uncomplicated pregnancies exercise before, during, and after pregnancy [46,47]. According to the ACOG, few pregnant women, including those who are categorized as overweight and obese, are meeting current physical activity guidelines [48]. However, the reasons as to why women are not meeting guidelines remain unclear. Our findings show that among pregnant and postpartum women, before commencing their pregnancies, both groups indicated that they planned on exercising during pregnancy. Albeit, when asked whether they exercised as much as they would have liked throughout pregnancy, most women indicated “No.”

For currently pregnant women, barriers related to physical discomfort, contraindications to exercise communicated by HCP, and self-perception were associated with a reduced likelihood of meeting IOM guidelines whereas, for postpartum women, barriers related to social support from family and friends were associated with a lower odds of meeting recommendations. A recent systematic review of qualitative and quantitative studies by Harrison and colleagues, on the attitudes, barriers, and enablers to physical activity during pregnancy found that intrapersonal factors (i.e. fatigue, lack of time, discomfort) served as barriers to physical activity whereas social support played an enabling role during pregnancy [49]. Similarly, our findings indicate that women reported intrapersonal factors as barriers to achieving a healthy pregnancy weight gain and a supportive social environment as a significant facilitator to gestational weight gain guideline adherence.

We also demonstrated that most women recognize the importance of exercising during pregnancy. The perceived attitude that exercise is essential may influence future behaviour (i.e. physical activity) in agreement with the Theory of Planned Behaviour mentioned earlier [36]. Though, despite positive attitudes towards exercise, women continue not to meet physical activity guidelines during pregnancy. What is more, interventions based on goal setting strategies have proven useful in preventing excessive GWG [24]. As such, a plan to exercise during pregnancy and self-monitoring strategies may be the first necessary step to achieving optimal weight gain during pregnancy and to remain physical activity throughout. Given that physical activity during pregnancy plays a protective role against excessive GWG [50] and is associated with numerous psychological benefits such as reduced anxiety, depression, fatigue, and overall improved well-being [51], future interventions must consider intrapersonal factors and social support, along with goal setting strategies as a means of encouraging women to engage in physical activity prior to, during, and after pregnancy.

### Limitations and strengths

There are important limitations and several strengths worthy of mentioning. Self-reporting and recall bias were possibilities since the survey was self-administered. Importantly, our survey validation study [27] stated that following pregnancy, women are able to accurately recall pregnancy-related characteristics and behaviours over 30 years, which include pre-pregnancy height and weight, obsetric complications, birthweight, and GWG within one year of delivery and 4 to 12 years after pregnancy. Furthermore, a study that concluded overweight and obesity is associated with excessive GWG, found that this was not dependent on self-reported weight status since approximately 20% of women with overweight or obesity accurately identified their weight status [52]. It is also important to note that this study sample was composed of predominantly educated women who received prenatal care in Canada or the USA, were employed, married, between the ages of 30 to 39, and of high socioeconomic status. Although this sample may not be representative of the diverse global population, understanding novel psychosocial factors such as weight-related self-efficacy for meeting American IOM guidelines can help guide clinical practice and future interventions aimed at reducing excessive GWG in industrialized nations. Lastly, another limitation is that our study relied on cross-sectional data, dismissing causal attributions. Therefore, the relationship between meeting IOM guidelines for weight gain during pregnancy and the correlates presented in this study are strictly associations.

A considerable strength of this study is the large sample size of pregnant women and new mothers. In addition, the EMat health survey allowed for a complete analysis of barriers and facilitators surrounding the psychology of women’s health during pregnancy. Given that the survey is grounded in SCT, this allowed for understanding of how self-efficacy and locus of control contribute to healthy pregnancy weight gain. Many studies to date have failed to discover an association between psychological factors and GWG or neglect to examine these factors altogether [20,40]. Previous reviews have identified similar findings with respect to attitude towards weight gain during pregnancy and the protective effect of internal locus of control over weight gain [21,22]. This study has identified significant psychosocial risk factors associated with excessive GWG. However, it is essential to recognize the complexity of psychosocial factors associated with excessive GWG. At a time where excessive GWG has reached epidemic proportions, studies such as these that can help elucidate psychological and social contribtors to weight gain outside of guidelines, will help develop adequate interventions to meet the immediate and long-term health needs of pregnant women and their offspring.

## Conclusion

The findings from this study contribute to the growing literature on deciphering what risk factors are associated with excessive GWG. Firstly, we discovered that a high self-efficacy, associated with an internal locus of control served a protective role against exceeding IOM weight gain recommendations, whereas low self-efficacy and an external locus of control diminished the likelihood of meeting IOM guidelines. Secondly, pregnant women who described intrapersonal factors as barriers to exercise while pregnant and postpartum women who depicted social support/environment were significantly less likely to achieve guideline-concordant weight gain. Future work should investigate interventions that combine HCP guidance and consideration for psychosocial factors during prenatal care, to ultimately contribute towards healthier pregnancies and future generations.

## Supporting information

S1 Dataset De-identified dataset from the Electronic Maternal health survey. (XLSX)
